# Essays for the Columbian Congress

**Published:** 1892-11

**Authors:** 


### Editorial.

### ESSAYS FOR THE COLUMBIAN CONGRESS.

The committee of the World's Columbian Dental Congress having in charge the selection of papers to be read before that body have issued their preliminary circular, which will be found on another page of this number. This, in our opinion, is the most important committee of that congress; for upon it will rest the responsibility for the character of the meeting.

It would be very ill advised to give an opinion in advance of what this committee may or may not do; but some general ideas may not be out of place in this connection. The "stream cannot rise higher than the fountain," and the source, in this case, lies in the dentists who propose to enlighten this body. To them, therefore, a word may be of value, and perhaps be some aid in eliminating prolixity, and thus relieving the committee from a useless waste of time and the writers from a possible rejection of their manuscripts.

The decision as to the acceptance or non-acceptance of an essay we are authorized to state will be mainly based on its scientific value. The sentence in the circular which reads, "Those contributions earliest received will, of course, take precedence," seems to give a different idea, and may possibly lead to the inference that papers are to be selected indiscriminately. This is not to be the rule, and should not be.

This congress will, without doubt, be remarkable in many directions. Numbers are assured, but mere size amounts to but little, and in time will hardly remain even as a memory. Distinguished men from all parts of the world will be at the head and among the active participants, but great men pass away and are forgotten. There is but one thing that can live in a convention of this character, and that is the subject-matter evolved from the combined mental force present in its deliberations and active in its work. This will best be shown in the essays presented and in the discussions. Original scientific matter will continue to exist and cause this World's Dental Congress to be remembered when the men who originated it are forgotten. Platitudes, insipidity, verbiage, all deserve to be strangled in their birth, and hence cannot be expected to pass the criticism of the committee.

It is a well-known fact that those best qualified to perform scientific labor of value in their several lines of thought very generally neglect it. There is a large contingent in the United States who, no doubt, are revolving in their minds original work, but unless forced to see the necessity of beginning the preparation at once will defer it until too late to make it available. As we stated in a former number, now is the time to prepare. The papers must contain original matter in order to command respect. Padding by quotations should be avoided, as the time of the congress is too valuable for anything but the subject in hand. The personal pronoun "I" should cease to be a prominent feature in the arrangement of sentences. These should be confined to the relation of facts. Let the paper be read daily for a week, cutting out all superfluous matter, and finally, after this careful revision, have it type-written into at least three copies.

This convention has a much higher object than merely bringing together men from all parts of the world as an excuse to help build up the great Fair. Dentistry in America will be on trial, and we have invited all the nations to come and see what is really meant by the term "American Dentistry." It has been claimed, and we think justly, that the workers in this profession have made it what it is to-day. It is necessary that this bold assumption should be made good. To do this means something more than will be possible to manifest at Chicago. The examination should cover our whole system of instruction. Our colleges should be visited. The large establishments for the manufacture of dental materials should all be open for inspection. Nothing should be kept secret. Let all doors be swung upon their hinges and the world invited to enter the most protected of inner sanctuaries, if such exist.

The charge has been made repeatedly by our European confrères that, while they are perfectly willing to accord to America—and by this they mean the United States and Canada—the credit of being in the lead in mechanical matters, we are deficient in those things that go to make a profession, theoretical attainments in collateral medical branches. Whether this be true or not, it is fully and honestly believed in the Old World, and the sneer of contempt is not infrequently hurled at us as though we were in the age of scientific childhood. It is not so important to disabuse prejudiced minds as it is to develop a national self-respect, and this can be accomplished only by direct comparison. This privilege will be accorded at this convention as never before, and if not decided in our favor, then we must be satisfied to rest on the laurels we have earned as most excellent mechanicians and inventors.

The hours that intervene between now and the realization of the work of this congress will be periods of anxiety and labor. To relieve this, in a measure, let every scientist in our calling resolve to do his part to lighten the work of the committee, and aid in making this convention worthy the esteem of all peoples.

